# Scaling Up Maternal Mental healthcare by Increasing access to Treatment (SUMMIT) through non-specialist providers and telemedicine: a study protocol for a non-inferiority randomized controlled trial

**DOI:** 10.1186/s13063-021-05075-1

**Published:** 2021-03-05

**Authors:** D. R. Singla, S. E. Meltzer-Brody, R. K. Silver, S. N. Vigod, J. J. Kim, L. M. La Porte, P. Ravitz, C. E. Schiller, N. Schoueri-Mychasiw, S. D. Hollon, A. Kiss, D. Clark, A. K. Dalfen, S. Dimidjian, B. N. Gaynes, S. R. Katz, A. Lawson, M. Leszcz, R. G. Maunder, B. H. Mulsant, K. E. Murphy, J. A. Naslund, M. L. Reyes-Rodríguez, A. M. Stuebe, C-L Dennis, V. Patel

**Affiliations:** 1 Department of Psychiatry, Sinai Health and Lunenfeld Tanenbaum Research Institute, Toronto, Canada; 2 Department of Psychiatry, Faculty of Medicine, University of Toronto, Toronto, Canada; 3 Department of Psychiatry, School of Medicine, University of North Carolina, Chapel Hill, NC USA; 4 Department of Obstetrics & Gynecology, NorthShore University HealthSystem, Chicago, IL USA; 5 Women’s College Hospital and Research Institute, Toronto, Canada; 6 Department of Obstetrics & Gynecology, University of Chicago Pritzker School of Medicine, Chicago, USA; 7 Department of Psychology, Vanderbilt University, Nashville, TN USA; 8 Sunnybrook Research Institute, Toronto, ON Canada; 9 Department of Experimental Psychology, University of Oxford, Oxford, UK; 10 Renee Crown Wellness Institute and Department of Psychology and Neuroscience, University of Colorado, Boulder, CO USA; 11 Department of Obstetrics and Gynecology, Sinai Health and University of Toronto, Toronto, Canada; 12 Centre for Addiction and Mental Health, Toronto, Canada; 13 Department of Global Health and Social Medicine, Harvard Medical School, Boston, MA USA; 14 Department of Obstetrics & Gynecology, School of Medicine, University of North Carolina, Chapel Hill, USA; 15 Lawrence S. Bloomberg Faculty of Nursing and Department of Psychiatry, University of Toronto, Toronto, Canada; 16 Li Ka Shing Knowledge Institute, St. Michael’s Hospital, Toronto, Canada; 17 Department of Global Health and Population, Harvard T.H. Chan School of Public Health, Boston, MA USA

**Keywords:** Depression, Anxiety, Psychological treatments, Behavioral activation, Telemedicine, Perinatal, Randomized controlled trial

## Abstract

**Background:**

Depression and anxiety impact up to 1 in 5 pregnant and postpartum women worldwide. Yet, as few as 20% of these women are treated with frontline interventions such as evidence-based psychological treatments. Major barriers to uptake are the limited number of specialized mental health treatment providers in most settings, and problems with accessing in-person care, such as childcare or transportation. Task sharing of treatment to non-specialist providers with delivery on telemedicine platforms could address such barriers. However, the equivalence of these strategies to specialist and in-person models remains unproven.

**Methods:**

This study protocol outlines the Scaling Up Maternal Mental healthcare by Increasing access to Treatment (SUMMIT) randomized trial. SUMMIT is a pragmatic, non-inferiority test of the comparable effectiveness of two types of providers (specialist vs. non-specialist) and delivery modes (telemedicine vs. in-person) of a brief, behavioral activation (BA) treatment for perinatal depressive and anxiety symptoms. Specialists (psychologists, psychiatrists, and social workers with ≥ 5 years of therapy experience) and non-specialists (nurses and midwives with no formal training in mental health care) were trained in the BA protocol, with the latter supervised by a BA expert during treatment delivery. Consenting pregnant and postpartum women with Edinburgh Postnatal Depression Scale (EPDS) score of ≥ 10 (*N* = 1368) will be randomized to one of four arms (telemedicine specialist, telemedicine non-specialist, in-person specialist, in-person non-specialist), stratified by pregnancy status (antenatal/postnatal) and study site. The primary outcome is participant-reported depressive symptoms (EPDS) at 3 months post-randomization. Secondary outcomes are maternal symptoms of anxiety and trauma symptoms, perceived social support, activation levels and quality of life at 3-, 6-, and 12-month post-randomization, and depressive symptoms at 6- and 12-month post-randomization. Primary analyses are per-protocol and intent-to-treat. The study has successfully continued despite the COVID-19 pandemic, with needed adaptations, including temporary suspension of the in-person arms and ongoing randomization to telemedicine arms.

**Discussion:**

The SUMMIT trial is expected to generate evidence on the non-inferiority of BA delivered by a non-specialist provider compared to specialist and telemedicine compared to in-person. If confirmed, results could pave the way to a dramatic increase in access to treatment for perinatal depression and anxiety.

**Trial registration:**

ClinicalTrials.gov NCT 04153864. Registered on November 6, 2019.

**Supplementary Information:**

The online version contains supplementary material available at 10.1186/s13063-021-05075-1.

## Introduction

### Background

Depression is a leading cause of disability worldwide [1] with an estimated 10 to 15% of women worldwide experiencing depression during pregnancy or the year following childbirth [2, 3]. Symptoms often begin in the antenatal period [4], and estimates of the annualized lifetime costs of perinatal depression amount to over $45.9 billion USD dollars in the USA alone [5]. Although given less attention than depression, up to 20% of mothers also experience anxiety symptoms [6] and approximately 10% are co-morbid for both [7]. The negative impact of these disorders on mother and child [8–10] underscores the importance of addressing perinatal mental health.

Psychological treatments, such as cognitive, behavioral, and interpersonal therapies, are first-line, effective interventions for perinatal depression and anxiety [11, 12] and are preferred by many women over pharmacotherapy [13, 14]. Despite the recent US Preventive Task Force Recommendations for evidence-based psychological treatments for perinatal populations [15], as few as 20% of affected women are treated with adequate treatments in North America [16]. Barriers include childcare needs, costs, transportation, and stigma [13, 17], in addition to the paucity and inequitable distribution of mental health professionals across settings. Given their prevalence and detrimental impact, developing low-cost, innovative solutions to improve access to psychological treatments for perinatal depression and anxiety is a public health priority. This is particularly true in the context of the COVID-19 pandemic and its impact on mental health symptoms.

Task-sharing is used worldwide to improve access to care, with non-specialist providers (NSPs)—individuals with no formal training in delivering mental health care such as nurses, peers, lay counselors, midwives, teachers, and primary care doctors—trained to effectively treat perinatal depressive and anxiety symptoms [18, 19]. NSPs are widely available, affordable, and often have regular contact with women in the course of their healthcare during pregnancy and the postpartum period [18, 20, 21]. NSPs have effectively delivered behavioral activation (BA) to reduce maternal depressive symptoms [22, 23], anxiety symptoms [23], and intimate partner violence among women of childbearing age [22]. In high-income countries, nurses and midwives are among the most likely to deliver effective psychological interventions [24] and appear to be a preferred type of NSP by perinatal women [25]. Whether specialists and NSPs are equally effective in targeting perinatal depressive and anxiety symptoms when delivering the *same* treatment remains unknown.

Telemedicine-based psychological treatments offer an alternative approach for perinatal women in terms of flexibility [26], efficiency [27], and cost [28]. This delivery method also increases accessibility and scalability. Recent reviews suggest that telemedicine is as efficacious as in-person psychotherapy on depression and anxiety are similar [29, 30]. However, most of these studies have not compared in-person visits to telemedicine platforms and are underpowered to establish equivalence of the two treatment formats [30].

BA proposes that the key to reducing depression and anxiety is to increase enjoyable or fulfilling activities that align with one’s values [31, 32], targeting key mechanisms of patient activation and avoidant coping. BA has strong evidence for its effectiveness in treating depression in the general population [33, 34]. A randomized placebo controlled study in the USA found that BA was as efficacious as, and more enduring than, antidepressant medication, with fewer patients dropping out of treatment [35, 36]; in the USA and UK, BA was as effective as longer courses of cognitive behavioral therapy [36, 37]. BA was also as effective in treating perinatal depression in randomized trials in the USA, where BA was associated with high satisfaction and treatment engagement [21, 38] and offered significant benefit compared with treatment as usual [23, 39]. Further, BA is easy to understand and implement [31], which is critical when training NSPs. Trials in the USA [23], Uganda [40], India and Nepal [22, 41, 42], and the UK [43] have demonstrated that lay counselors, psychology undergraduates, maternal peers and midwives can all be trained to deliver BA to reduce depressive symptoms.

### Primary objectives

The primary objectives of this study are twofold:

1. To examine if BA can be delivered as effectively by NSPs as specialists in treating perinatal depressive symptoms, as measured by the Edinburgh Postnatal Depression Scale (EPDS) [44]; and
2. To examine if BA delivered through telemedicine is as effective as BA delivered through in-person format, in treating perinatal depressive symptoms as measured by the EPDS [44].

### Secondary objectives

1. To examine the aforementioned questions on anxiety symptoms, as measured by the Generalized Anxiety Disorder-7 (GAD-7) [45].
2. To assess moderating effects of clinical severity and treatment timing (antenatal vs. postnatal) on the comparative effectiveness of the two delivery formats on depressive and anxiety symptoms;
3. To determine whether timing of the treatment influences child mental development at 9 to 15 months post childbirth; and
4. To describe the underlying processes, barriers, and facilitators to the delivery of BA for perinatal depressive and anxiety symptoms from a multi-stakeholder perspective.

The COVID-19 pandemic has amplified the need for effective mental healthcare and led to a dramatic increase in the use and role of telemedicine platforms [46]. While following key guidelines for preserving clinical trial integrity, the study has continued during the pandemic [47]. A list of adaptations made to study implementation is presented in Table 5.

## Methods—design, setting, participants, interventions, and outcomes

### Trial design

This is a multi-center, four-arm, randomized, non-inferiority trial to test the comparable effectiveness of delivery mode (telemedicine vs. in-person) and provider (NSP vs. SP), implementing a brief, evidence-based BA treatment for perinatal depressive and anxiety symptoms. In this trial, we will also determine the underlying processes related to delivery of this brief psychological intervention from a multi-stakeholder perspective. The protocol conforms to the Standard Protocol Items: Recommendations for Interventional Trials (SPIRIT) guidelines (see Additional file 1: Figure S1 and Additional file 2: SPIRIT checklist).

### Setting

The study takes place in three hubs—Toronto, Chapel Hill, and Chicago. In Toronto, recruitment is at Sinai Health (SH), Women’s College Hospital (WCH), and St. Michael’s Hospital (SMH)—three academic hospitals affiliated with the University of Toronto. In North Carolina, we are recruiting from three clinical sites affiliated with the University of North Carolina (UNC) Women’s and Neuroscience Hospitals. In Chicago, we are recruiting from fourteen affiliated obstetric and family medicine clinics affiliated with NorthShore University HealthSystem and the University of Chicago.

### Participants and procedures

**Fig. 1:**
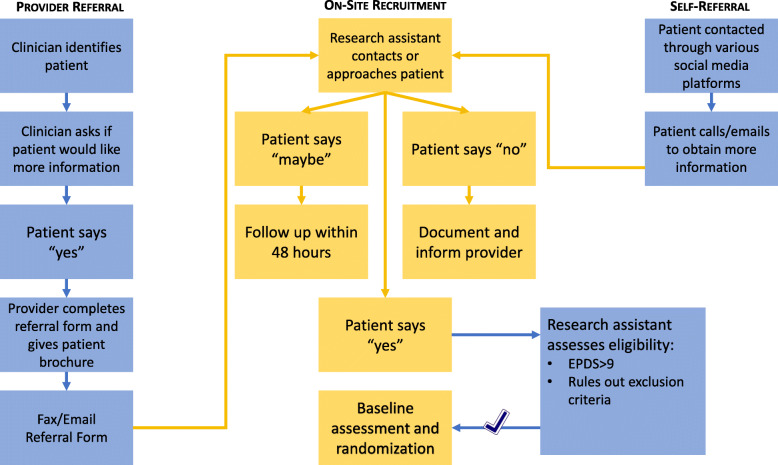
Recruitment pathways

We plan to recruit 1368 pregnant and postpartum women aged ≥ 18 (Fig. 1). Inclusion and exclusion criteria are detailed in Table 1. The study is introduced to eligible women by a trained research assistant or one of the women’s clinical providers at their respective study site. Recruitment and informed consent are acquired in-person or via telephone, using Research Electronic Data Capture system (REDCap™). After consenting to participate, women are screened on the Edinburgh Postnatal Depression Scale (EPDS ≥ 10 [44]) for eligibility. A systematic effort is made to recruit ethnically diverse populations, including women from Latinax communities. For example, bilingual research assistants at the US sites introduce the study in Spanish with a translated consent form. In-person study activities have been paused since the onset of the COVID-19 pandemic (see Table 5).

**Table 1:** Inclusion and exclusion criteria

**Inclusion**	**Exclusion**
• EPDS ≥ 10 • ≥ 18 years • Pregnant up to 36 weeks or 4 to 30 weeks postpartum • Speaks English or (US sites) Spanish	• Active suicidal intent (ideation and plan), active symptoms of psychosis or mania • Psychotropic medication dose or medication change within 2 weeks of enrollment or beginning treatment • Ongoing psychotherapy • Active substance abuse or dependence • Severe fetal anomalies, stillbirth, or infant death at time of enrollment for index pregnancy • Non-English, non-Spanish speakers

### Treatment and intervention arms

#### Treatment

The SUMMIT treatment consists of six to eight individual weekly BA sessions. The manual was adapted from two well-established source manuals—the Alma Program for perinatal populations in Colorado and the Healthy Activity Program (HAP [22, 48];) from Goa, India. Key strategies include: psychoeducation, behavioral assessment, values-based activity monitoring and structuring, interpersonal effectiveness, and problem solving. Unlike cognitive behavioral interventions for depression, BA explicitly targets avoidant coping [49] and has been effective in reducing anxiety symptoms [50].

#### Intervention arms

**Fig. 2:**
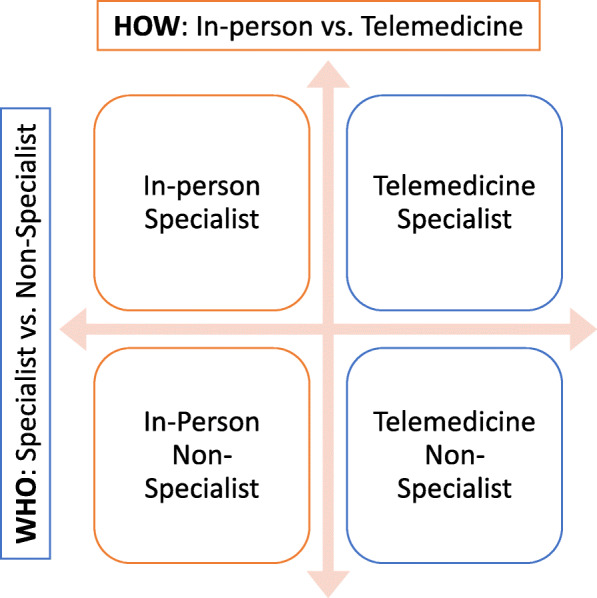
SUMMIT treatment arms

Participants are randomized to one of four arms (Fig. 2):

#### Non-specialists vs. specialists

SUMMIT treatment providers include both NSPs and specialist providers (SPs). NSPs are registered nurses (RNs) or midwives with general health care professional skills but without formal training in mental health care or experience delivering psychotherapies. SPs include individuals with formal training in mental health care delivery (e.g., psychiatrists, psychologists, and social workers) and with experience in treating mental illness and a minimum of 5 years of experience delivering psychological treatments.

#### Telemedicine vs. in-person

Telemedicine is implemented via the Ontario Telemedicine Network and Zoom™ in Toronto, the UNC TelePsychiatry Program in Chapel Hill, and Zoom™ in Chicago. All platforms (1) permit video-visits and scheduling; (2) are accessible on PC, Mac, Android and iOS systems; and (3) are PHIPA/HIPAA compliant. For participants lacking access to a technology, study tablets are provided for use on a temporary basis. Participants assigned to telemedicine can conduct BA sessions in a preferred private location (e.g., home). In-person sessions are held at participating clinical care sites (paused during COVID-19).

### Recruitment, training, supervision of treatment providers

The same procedures were implemented in each hub.

#### Recruitment

All NSPs were recruited through advertisements in relevant listservs and word of mouth and selected based on their performance in a structured interview and role-play during an intake interview. A mock patient was incorporated and the Therapy Quality Scale was used to rate the potential provider [51]. SPs were recruited through advertisements and word of mouth. They were selected based on location, interest and availability, and good standing with their respective colleges.

#### Training and competency tests

Separate workshops were held to train NSPs and SPs. A minimum of two clinical leads conducted each training workshop to ensure consistency across hubs. Clinical leads are expert clinicians and co-Is (PR in Toronto, CS in Chapel Hill, and JJK and LML in Chicago) who are designated to oversee the training and clinical implementation of the BA treatment along with supervision. The workshops utilized observation and didactics with interactive educational strategies including role play, games, and homework. The primary objectives of the training workshops (see Table 2) were to prepare the providers to implement the BA manual and SUMMIT protocol with its safety procedures, including when to refer a participant for post-trial care. Trainees meeting competency standards (based on a multiple-choice questionnaire [52] and in an interaction with a standardized patient (actor) rated by experts using an adapted version of the Quality of HAP (Q-HAP scale) [51] were selected for the 8-week internship phase of the trial. During this phase, trainees saw up to two participants (one via telemedicine and one in-person) to implement the BA treatment. Only trainees who achieved competence, as assessed by standardized role-plays and therapy quality assessments, were selected to deliver BA during the trial.

**Table 2:** Training outline by provider

	Non Specialist Provider	Specialist Provider
Day 1	• Introduction to the trial • Background in perinatal depression and anxiety • Mood measures (EPDS + GAD-7) • Foundational skills for therapy: EMPOWERS • Introduction to the principles of BA • BA: session 1	• Introduction to the trial • Mood measures (EPDS + GAD-7) • Introduction to the principles of BA • BA: sessions 1–2
Day 2	• BA: sessions 2–3	• BA: sessions 3–8
Day 3	• Safety protocols	• Safety protocols • Multiple choice exam • Competency role-play • Training evaluation
Day 4	• BA: sessions 4–8 • Multiple choice exam • Competency role-play • Training evaluation	—

#### Supervision

During the training phase, clinical leads provided weekly supervision with NSPs and SPs (separately to avoid contamination) to review and discuss cases and reinforce BA training. All treatment providers recorded all BA sessions for training and supervision purposes and for fidelity measures. Treatment providers who required additional assistance to gain competence completed an additional training case with additional supervision.

During the trial, NSPs have continued weekly supervision meetings with their designated clinical lead. In addition, NSPs engage in monthly measurement-based supervision where clinical leads and NSPs rate individual audio-recorded sessions for therapy quality—the extent to which a psychological treatment was implemented well enough to achieve its expected effects [53]. Audio recordings are rated using the 20-item Q-HAP [51] for BA treatment-specific and common therapeutic (e.g., collaboration, empathy) skills. The individual NSP who conducted the session (self-rating), 2–4 peers (peer ratings), and a clinical lead (expert rating) evaluate a selected session. The summed score by peers for each subscale is used to estimate therapy quality for the session and is compared to the expert ratings. As the trainee NSPs gain experience in delivering BA and rating audio-recorded sessions, the supervision format will evolve from expert led (that is, the clinical lead and specialist who is skilled in the delivery of the psychological treatment to peer-led group supervision) [51]. SPs do not attend regular supervision, but the clinical lead is available on an ad hoc basis to reflect real-world conditions.

### Fidelity assessments

Independent consultants evaluate up to 5% of all audio sessions by both NSPs and SPs to assess treatment adherence and fidelity during the trial phase. Treatment providers not reaching the cut-off for specific items will receive refresher training by the hub clinical leads.

### Outcomes and timing of assessments

Table 3 lists all measures and the timing of assessments. In general, there are measures related to the participant (measured at baseline, 3-, 6-, and 12-month post-randomization), the therapy (session-wise scores and end of treatment), and the participant’s child (assessed at 9–15 months post-childbirth). All measures (Table 3) have been validated and previously used in one or more of the investigators’ trials targeting perinatal mental health (e.g., [22, 23, 40, 41, 54, 55, 61, 68, 69]. The assessment period is extended if there is a treatment hiatus due to perinatal life events (e.g., giving birth, obstetrical complications, or contracting COVID-19).

**Table 3:** Outcomes and timing of assessments

Study variable	Instrument	Outcome (range)
***Maternal: measured at baseline and 3***^a^***-, 6-, and 12-month post-randomization***		
**Maternal characteristics**^b^	**Trial Baseline Questionnaire** [54, 55]	Self-reported age, education level, gender and sexual orientation, marital status, race, immigrant status and ethnicity, clinical history with depression or anxiety (severity, chronicity, number of prior episodes, and age at first episode), occupational status, number of children, pregnancy intention, pregnancy history, delivery, and birth.
**Depressive symptoms**	**Edinburgh Postnatal Depression Scale (EPDS)** [44]	Mean continuous score of a 10-item scale (0–30)
**Anxiety symptoms**	**Generalized Anxiety Disorder Scale (GAD-7)** [45]	Mean continuous score of a 7-item scale (0–21)
**Response and remission**	**Patient Health Questionnaire-9 (PHQ-9)** [56]	Response: PHQ < 10 Remission is defined as PHQ < 5
**Perceived support**	**Multidimensional Scale of Perceived Social Support** [57]	Mean continuous score of a 12-item scale (1–84)
**Disability assessment**	**World Health Organization Disability Assessment Schedule (WHODAS)** [58]	Mean continuous score of a 12-item scale (0–48)
**Quality of life assessment**	**EQ 5D-5 Level** [59]	Mean continuous score of a 5-item scale (1–25)
**Trauma symptoms**	**Abbreviated PTSD Checklist (PCL-6)** [60]	Mean continuous score of a 6-item scale (1–30)
**Patient-reported activation**	**Premium Abbreviated Activation Scale** [41, 61]	Mean continuous score of a 5-item scale (0–20)
**Patient satisfaction**^c^	**Client Satisfaction Questionnaire-8 (CSQ-8)** [62]	Mean continuous score of an 8-item scale (1–32(
**Therapeutic alliance**^c^	**Working Alliance Inventory-Short Revise (WAI-SR)** [63]	Mean continuous score of a 12-item scale (1–60)
**Health service utilization**	**Health Service Utilization Form**	Total score of a 16-item scale (0–32)
**Treatment preference**^b^	**Delivery of treatment and treatment provider preference**	Score of 0 or 1
**COVID-19 exposure**	**1-item question on COVID-19 exposure**	Self-reported
***Treatment: measured at every session during treatment, unless otherwise indicated***		
**Dosage**	**Treatment log** [64]	Frequency of sessions attended
**Therapy quality**^d^	**Quality of Healthy Activity Program (Q-HAP)** [51]	Mean continuous score of treatment-specific BA skills (0–4) and general counseling skills (0–4)
**Session depressive/anxiety**	**Session-by-session EPDS** [44] and **GAD-7** [45] **scores**	Mean continuous score of a 10-item scale (0–30) on EPDS and of a 7-item scale (0–21) on GAD-7
**Homework adherence**	**Treatment log** [64]	Mean continuous score of a 1-item question (0–2)
**Adverse or serious AEs**	**Anytime an adverse event (AE) or serious AE occurs**	Any event that represents a series threat to the safety of the mother or her child
**Health service utilization**	**Health Service Utilization Form** [65]	Total score of a 16-item scale (0–32)
**List of medications**	**List of medications**	Self-reported list of medications
**COVID-19 exposure**	**1-item question on COVID-19 exposure**	Self-reported
***Child: measured at 9 to 15 months post-child birth unless otherwise indicated***		
**Birth weight and length**	**Retrieved from hospital chart or self-report**^e^	Assessed at birth
**Breastfeeding**	**Whether breastfeeding and if stopped age stopped** [55]**.**	Total number of months (0–12)
**Psychosocial stimulation**	**Home Observation Measurement Evaluation** [66]	Total score of a 45-item checklist
**Mental development**	**Bayley Mental Development Scales IV** [67]	Mean continuous score of cognitive, receptive, and expressive language development

^a^Assessment period will be extended to account for post-treatment outcomes when there are perinatal-related interruptions to treatment (e.g., giving birth, obstetrical complications, COVID-19)

^b^Only at baseline

^c^Measured at 3 months post-randomization only

^d^Randomly selected for supervision, rated by self, peers, expert supervisor

^e^Self-report will be used when hospital charts are outside of the recruiting site

### Sample size

The primary outcome measure is an EPDS mean score at 3 months post-randomization. The sample size calculation is based on an EPDS mean estimate of 7.93 (SD = 4.68 [54]). Using a non-inferiority margin of 10% (i.e., EPDS score of 0.79 in relation to the mean), and an alpha = 0.025 (i.e., 0.05/2 for our two key comparisons of interest: telemedicine vs. in-person and SP vs. NSP), we require 274 participants in each of the four arms to provide greater than 80% power. To account for 20% drop out, and provide additional power (up to 76%) for pairwise comparisons between the four arms (i.e., telemedicine SP, telemedicine NSP, in-person SP, in-person NSP) and 80% power for our secondary hypothesis of reduced anxiety (mean GAD-7 = 8.07, SD = 5.50 [23]), the required final sample is *N* = 1368 (342 per arm).

### Participant recruitment

Across the three hubs, we anticipate assessing 18,280 participants for potential eligibility and obtaining informed consent to screen 9140 from which we will recruit and retain a sample size of *N* = 1368 women (see Fig. 2). We anticipate that 50% of participants will be recruited in Toronto, and 25% each in Chapel Hill and Chicago.

#### Nested qualitative study

**Fig. 3:**
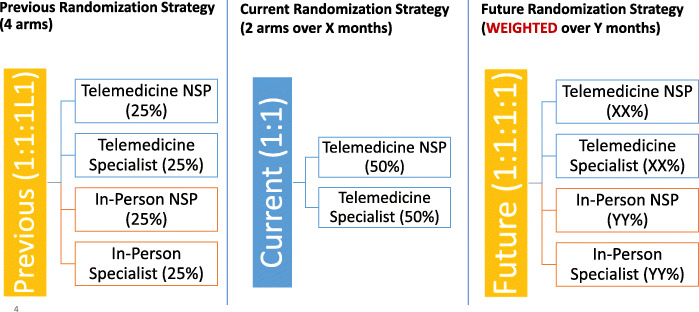
Study randomization and adaptation due to COVID-19

**Fig. 4:**
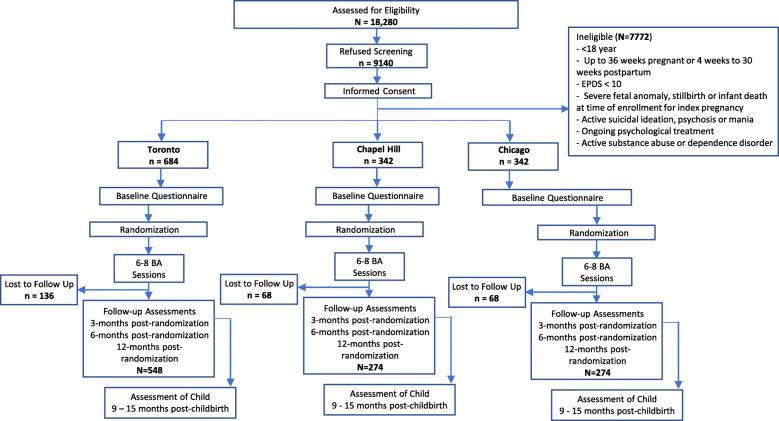
Study flow chart

Interviews are being conducted with various subsets of participants (*n* = 60) including representation from each of the four arms, treatment completers vs. non-completers, and antenatal vs. postnatal enrollment. A maximum variance sample will be used to capture participants across severity levels, perinatal periods, site key sociodemographic factors, and stakeholder groups. An unblinded data coordinator identifies potential participants. In addition, we are interviewing up to 20 significant others (including spouses or partners of participating mothers), all SPs and NSPs, 10 healthcare providers (obstetricians, family physicians, nurses, midwives, psychiatrists and psychologists not participating in the study), and relevant stakeholders such as patient advocates. Each treatment provider will be interviewed three times, once during each phase of their participation in the trial (beginning, middle, and end) to capture relevant aspects of the intervention during implementation. Finally, we will examine the data to determine the presence of specific barriers, facilitators, and modifications made to the content and delivery of the BA treatment in response to COVID-19 (Figs. 3 and 4).

## Methods—allocation, data collection, management, and analysis

### Randomization

After informed consent, verification of inclusion/exclusion criteria, and completion of baseline measures, participants are stratified by whether they enrolled antenatally or postnatally then randomized within site to one of the four arms (1:1:1:1) via computer-generated randomization through REDCap™. Since the start of COVID-19 containment efforts in all study jurisdictions, in-person arms have been temporarily suspended (see Fig. 2) to decrease the number of non-urgent visits to hospitals and clinics and to decrease the risk of exposure to COVID-19 for both study participants and treatment providers, in line with institutional guidelines. Pending the duration of the COVID-19 outbreak and its impact on the safety of in-person care, the return to a four-arm trial will include weighting of randomization toward in-person sessions to “balance” the number of individuals who are currently being randomized to telemedicine.

### Blinding and masking

Baseline assessments are completed as per participant preference either remotely or on site (paused during COVID-19) at time of recruitment via a secure REDCap™ link prior to randomization. Outcome assessments at 3-, 6-, and 12-month post-randomization are also completed via REDCap™. While participants and providers are aware of treatment allocation, research assistants conducting the recruitment, participant qualitative interviews, and child in-home 9–15 months post-birth assessments will remain unblinded (Fig. 1; Table 3).

### Data collection

All data are collected via REDCap™.

### Data management

#### Quantitative data

De-identified data are collected in identical REDCap™ databases kept on secure institutional servers within each of the three participating cities. De-identified data from the USA hubs are extracted from the hubs’ REDCap™ database, encrypted, and transferred to Toronto, where they are uploaded, entered, and stored in REDCap™.

#### Qualitative data

All study interviews and focus groups are audio-recorded using a digital voice recorder. Audio files are transcribed and identifiers removed during transcription.

### Statistical analyses

#### Primary analysis

The primary analyses will be run as per-protocol and intent-to-treat, with two-sided significance levels of *p* < 0.05. Demographic (ethnicity, age, marital status) and other baseline variables (e.g., severity and chronicity) will be checked for differences between study groups using descriptive statistics (means, standard deviations, proportions) and the associated statistical tests (*t* tests, chi-square tests). The primary outcome (EPDS scores at 3 months post-randomization for telemedicine vs. in-person, and SP vs. NSP) will be analyzed using non-inferiority *t* tests. One *t* test will be run to compare treatment modality (is telemedicine non-inferior to in-person). Another *t* test will be run to compare providers (is NSP non-inferior to SP). Non-inferiority will be assessed by seeing whether the 10% non-inferiority margin is contained in the upper bound of the confidence interval around the difference in EPDS scores (e.g., between telemedicine and in-person). A sensitivity analysis will examine potential differences in baseline and outcome depressive and anxiety symptom scores to determine whether participants recruited and received treatment during the COVID-19 outbreak differed significantly from the larger sample. Multiple imputation methods will be used for data missing at random, with the number of imputations reflecting the percentage of missing data (e.g., if 10% of data is missing, then 10 imputations will be conducted) [70] and using SAS’s Proc MI and Proc MIANALYZE. Reasons for dropout will be ascertained from clinician records or by interviewing a subset of the participants who dropped out and coded. Drop-out characteristics will be compared to those completing the study. Sensitivity analyses will be carried out should missing data lead to the use of multiple imputation methods. These analyses will compare the results of the models on the imputed data to the ones with the actual missing data included. All analyses will be carried out using SAS Version 9.4 or later (SAS Institute, Cary, NC, USA).

#### Secondary analyses

In our secondary analysis, we are interested in (i) assessing the trend in EPDS and GAD-7 scores over time (baseline, 3-, 6-, and 12-month post-randomization) between groups (telemedicine vs. in-person, and SP vs. NSP). These analyses will use linear mixed models and include a group by time interaction term as well as adjust for potential covariates of interest. All child outcomes, including child mental development and the provision of psychosocial stimulation by the mother, will be compared between two groups (antenatal vs. postnatal) at 9–15 months post-childbirth using a two-sample two-sided *t* test. Sensitivity analyses will be carried out excluding those who dropped out after session one to see if the results are comparable to the entire study group.

### Moderation analyses

#### Clinical severity

Tests of moderation will be conducted to determine whether delivery mode (telemedicine vs. in-person) or agent (SP vs. NSP) is moderated by baseline clinical severity. We expect up to 15% of our sample to be severely depressed (EPDS > 20 [71]). A 2-group comparison with 75 per group and an assumed mean EPDS score of 20.0 (SD = 7.0)^86^, will provide 80% power, with an alpha of 0.05, to detect a mean change of 2.3 or greater. Our current sample size including those within severity subgroups described above will be adequately powered to detect medium effect size differences of heterogeneity of treatment effects of symptom severity between groups. This indicates that we have adequate power for clinically meaningful tests. The two groups’ (SP vs. NSP) EPDS scores at 3 months will be compared using a two-sided two-sample *t* test. Change in EPDS score over time (baseline and every 3 months post-randomization) will also be compared using linear mixed models. We will also examine this for anxiety symptoms at 3 months post randomization.

#### Perinatal period

This model will also test whether expectant mothers who receive antenatal treatment benefit more in terms of reduced depressive symptoms than mothers who receive postnatal treatment at 12 months post-randomization. A 2-group comparison with 200 per group and an assumed EPDS mean of 7.93 (SD = 4.68) will provide 90% power, with an alpha of 0.05, to detect a mean change of 1.5 points. This allows us to detect a drop corresponding to a small effect size of 0.3 (i.e., a drop to mean 6.43 on the EPDS). The two groups’ (antenatal vs. postnatal) EPDS scores at 3 months will be compared using a two-sided two-sample *t* test. Change in EPDS scores over time (baseline and every 3 months post-randomization) also will be compared using linear mixed models including the interaction between group and time. We will examine anxiety symptoms at 12 months post randomization in a linear mixed model controlling for relevant covariates including child age.

#### Child outcomes

We will examine whether the subset of mothers (up to 75% of the sample) who receive antenatal treatment benefit more in terms of improved child outcomes at 9–15 months than mothers who receive postnatal treatment. A 2-group comparison of 238 per group (antenatal vs. postnatal) and an assumed mean on any Bayley IV raw subscale score of 100, SD = 15 to assess child mental development will provide 80% power, with an alpha of 0.05 to detect a mean clinically-significant change of 3.0 units. Raw and standardized Bayley IV scores of the two groups’ children (antenatal vs. postnatal) at 12 months will be compared using a two-sided, two-sample *t* test.

#### Qualitative analysis

All qualitative data will be analyzed using NVivo™. We use content analysis with data analysis (coding) conducted by multiple independent raters, for whom inter-rater reliability is calculated using Kappa (κ) scores. Coding is conducted in a step-wise fashion to facilitate iterative revision and finalization of a coding scheme. Specifically, we first independently code and then discuss a minimum of 3 cases per stakeholder group to achieve a kappa (*κ*) score of *κ* = 0.75 or higher (defined as substantial to almost perfect agreement). Qualitative data is then quantified and triangulated across stakeholder groups using our previously established methods [20, 72].

### Drop out and follow-up strategies

Participants can withdraw consent and end their participation in the study at any time. We expect 20% of participants to be lost to follow-up. This dropout rate is conservative and has been found in many similar trials using NSP and SP delivered psychological treatments for perinatal depression in the USA and Canada [23, 39, 54, 55]. Based on a Cochrane review [73], prior research on study retention (e.g., [74–76]), and our previous experiences [40, 41, 54, 55], it is realistic to expect a 12-month post-randomization follow-up rate of at least 80% when we incorporate numerous retention strategies, such as questionnaire reminders and remunerations for their completion, obtained secondary contact information from participants, and being flexible with participants’ schedules.

## Oversight and monitoring

### Trial management

Several committees monitor the progress of the trial (Table 4). Trial monitoring comprises collation and reporting of routine trial process indicators and adverse events. The Data and Safety Monitoring Board (DSMB) consists of a psychiatrist with expertise in perinatal depressive or anxiety symptoms, a medical provider with expertise in providing obstetric care for pregnant women, a psychologist with expertise in the design and implementation of pragmatic clinical trials, and a PhD-level statistician. None of these persons are directly involved in the trial. The Trial Management Committee (TMC) presents regular reports to the Advisory Committee and the DSMB.

**Table 4:** Study management and committees

Committee	Role	Members	Frequency
Trial Management Committee (TMC)	To monitor all aspects of the conduct and progress of the trial, ensure that the protocol is adhered to and take appropriate action to safeguard participants and the quality of the trial.	• Principal investigator from each hub^a^ • Trial coordinators • Project administrator • Data coordinator	Weekly^b^
Investigator and Advisory Committee	To monitor all aspects of the conduct and progress of the trial, including site-specific safety protocols within and across sites	• All investigators • Research coordinators from each site • Data coordinator	Biweekly to Monthly
Stakeholder Advisory Committee (SAC)	To provide overall supervision of the trial and ensure that it is being conducted in accordance with the protocol and the relevant regulations. The TMC should approve the trial protocol and any protocol amendments and provide advice to the TMC on all aspects of the trial. Decisions about continuation or termination of the trial or substantial amendments to the protocol are finally the responsibility of the TMC.	• Members of the TMC • All investigators • Trial/content advisors and consultants • All stakeholders	Six-monthly
Data Safety Monitoring Board (DSMB)	The DSMB will review the accruing trial serious adverse event reports to assess whether there are any safety issues that should be brought to participants’ attention or any reasons for the trial not to continue. It is the only body that makes recommendations to unblind data and makes further recommendations to the TMC.	• Four members (clinical psychologist, obstetrician, perinatal psychologist, a statistician) with expertise randomized controlled trials assessing psychological treatments and perinatal populations	Six-monthly

^a^Hub refers to locality (Toronto, Chapel Hill, Chicago)

^b^ Weekly meetings also held within sites

### Stakeholder engagement and dissemination strategies

The SUMMIT research team works closely with a range of stakeholder partners across North America to inform the development, implementation, and dissemination of the SUMMIT trial. Two key goals include ensuring ample and appropriate stakeholder engagement as well as the delivery of diverse and audience-centered dissemination strategies.

To achieve the first goal of stakeholder engagement, three key steps are being implemented: (i) the creation of a diverse and informative Stakeholder Advisory Committee; (ii) identify and address potential barriers to engagement and implementation; and (iii) conduct an ongoing process evaluation to ensure that the treatment content, training materials and study questionnaires are acceptable and feasible from a multi-stakeholder perspective. Our stakeholder partners are listed on our study website (www.thesummittrial.com) and include individuals with lived experience, patient advocates and community partners, telemedicine partners, clinicians from various professions (nurses, midwives, family practitioners, obstetricians, social workers, psychologists, and psychiatrists), representatives from US-based insurance companies, and policy makers.

To achieve the second goal of audience-centered dissemination strategies, we work with our stakeholders to ensure that our dissemination strategies are inclusive and accessible to a wide range of networks. They include local, provincial/state-, national, and international methods of dissemination, involving press releases, presentations to local organizations and digital brochures, policy briefs, list-serves, and attending scientific meetings and publication of academic and non-academic publications.

### Serious adverse events (SAEs)

SAEs in this trial include disability or permanent harm, hospitalization for psychiatric reasons, life threatening events in the mother, fetus, neonate or infant, fetal or infant death, major congenital anomaly or birth defects, maternal death, or other serious important medical events such as active suicidal or homicidal ideation. Within 72 h of an SAE, the TMC reports the event to the DSMB, including details of the event, severity of any reactions, phase of the study, and procedures for its resolution, and reports to the Institutional Review Board of record.

The DSMB may also recommend termination or modification of the study in the following conditions: if SAEs are significantly clustered in one or more conditions as determined by the DSMB, followed by an unblinded analysis of all sites where we see patients worsening on depressive and anxiety symptoms by two or more standard deviations from the mean or based on the clinical judgment.

### Potential risk and benefits

Participants may or may not benefit from the study. The United States Preventive Task Force suggested that there is no-to-minimal harm of evidence-based psychological therapies [15]. If discussion of symptoms causes psychological discomfort or participants experience an exacerbation of depressive or anxiety symptoms, study safety protocols offset these risks. A treatment provider may experience distress if they are excluded from study participation for not meeting competency standards. In addition, NSPs may experience stress while participating as providers owing to their limited experience in this new role. These issues can be discussed at supervision. In addition, NSPs receive education about techniques in self-care as part of the BA training curriculum.

### Access to data

A data coordinator oversees the intra-study data sharing process, with input from the Trial Management Committee and trial Biostatistician. The trial Biostatistician has access to cleaned data. Hub PIs have direct access to their own, blinded site’s data and may have access to other sites’ data by request. The datasets analyzed during the current study will be available within 1 year of completing the trial from the corresponding author and upon reasonable request.

### Confidentiality

Data dispersed to relevant team members are always blinded to participant identity and treatment allocation, except for recruitment RAs, RAs who schedule appointments, the data coordinator, and treatment providers who can access relevant identifying information (e.g., session-wise EPDS and GAD-7 scores). All information is kept in password-protected, encrypted files on encrypted computers and secure servers. Unique study IDs are used to identify the participants with key files encrypted and stored on an encrypted computer or a secure server at each site.

## Discussion

This trial is designed to compare the clinical benefits of who (NSP vs. SP) and how (telemedicine vs. in-person) effective psychological treatment for perinatal depression and anxiety can be delivered. Our non-inferiority design and analytical strategy will determine if NSPs and telemedicine are comparable to the current standard of usual care (SPs and in-person). Our process evaluation will illuminate relevant barriers and facilitators related to training, supervision, intervention content and delivery across a wide range of stakeholders across several time-points of this trial.

As the largest psychological treatment trial for perinatal populations to date, SUMMIT’s findings have great potential to improve access to mental healthcare to address the growing burden of perinatal depression and anxiety. Examining these comparisons has the capacity to support a stepped care model in which we optimize the use of available resources. Continuing and adapting our study during COVID-19 (see Table 5) will allow us to produce results that are relevant to the current and future context of delivering psychological treatments with a patient-centered focus and the highest ethical standards. By reconceptualizing the delivery of psychological treatments in clinical practice, we can rethink the delivery of mental healthcare to implement sustainable, equitable, and patient-centered solutions. In sum, this research holds promise to improve the accessibility and scalability of brief, evidence-based, psychological treatments for perinatal women worldwide.

**Table 5:** Summary of COVID-19 related adaptations and lessons learned

	Changes to research processes	Facilitators and lessons learned
**Moving virtual**	All study procedures have transitioned to a secure and patient-centered **virtual system**, including consent, data collection, training and supervision and all follow-ups. E.g., • we are no longer asking participants to mail back signed consent forms during COVID-19. • Participants are giving verbal consent over the phone and via REDCap™, while research staff is keeping detailed logs of the process, or using e-signatures, depending on the site.	To facilitate this challenge, • **Materials are sent to participants electronically** with some exceptions pending the site-specific context. • **Guided participants** via phone and written instructions to ensure a timely and patient-centered approach in sending and receiving consent forms • **Ensuring regular communication** with trial participants and checking in with them to make sure they are completing surveys and/or address any questions that they may have.
**Study design and sensitivity analyses**	• Randomizing participants to in-person sessions was no longer acceptable or feasible at most sites • Randomization was temporarily modified to suspend in-person sessions and randomize only to telemedicine arms (see Fig. 2)	• **Expedited approvals** by our statisticians, DSMB, IRBs, and stakeholders • Our statistician will conduct **sensitivity analyses** to examine whether individuals who are exposed to this COVID-19 are potentially different from randomized to the study pre- and post-COVID
**Additional measures**	We have added the following measures: • **EQ 5D-5L** is used to measure quality of life • **PCL-6** measures trauma symptoms. Participants who have experienced previous trauma may experience exacerbated symptoms during COVID-19 • **Treatment preference** will be used to examine patient preferences in light of the recent, temporary modification of suspending in-person sessions	• Due to collecting new outcome data after a small group of participants had already been recruited, there will be a small portion of trial participants (*n* = 28 at baseline; < 2% of overall sample size) who do not have data for the new measures, and this will be accounted for as missing data during statistical analyses. • Additional measures will improve the scientific and pragmatic nature of the overall study and its potential implications for assessing these outcomes during COVID-19. These measures will allow us to compare participants’ experience during and after COVID-19.
**Recruitment**	In-person recruitment has been paused to uphold recommended social distancing guidelines for both participants and research staff	• All sites had **established referral pathways** with psychiatry and OB clinics prior to COVID-19, and once cold-recruitment paused, recruitment continued through those referral pathways. • All sites strengthened their referral pathways once in-person recruitment was no longer an option.
**All research staff working remotely**	All research staff are **working remotely** according to institutional guidelines, and have obtained remote access to secure servers	• The study was considered ‘essential research’ by department and hospital leadership. Redeployment was negotiated with individual sites • We ensured all team members had what they needed to work effectively, such as remote access to secure servers, regular check-ins and headphones. • Treatment providers were instructed (via written instruction and Zoom training) how to securely store their telemedicine sessions on their encrypted laptops and upload to the secured server once they can log in remotely. • One study site switched from their EMR-based telemedicine platform to using WebEx, as they were facing many technical issues with the former. Using WebEx is easier but required rolling out a new plan and platform for the providers and RAs.

### Trial status

Recruitment commenced in January 2020 and is estimated to be completed by April 2023. Recruitment numbers are updated regularly on the study website (www.thesummittrial.com). Modifications to the protocol that impact study conduct, potentially benefit patients or affect their safety require a formal amendment to the protocol. Amendments are agreed upon by the TMC and approved by relevant IRBs prior to implementation. Amendments for safety concerns are also reviewed by the DSMB prior to implementation. The current protocol version (1.0) was approved on December 18, 2020.

## Supplementary Information

**Additional file 1: Figure S1.** The Spirit flow diagram: the schedule of enrollment, intervention and assessments.

**Additional file 2.** SPIRIT checklist.

## Data Availability

Individuals interested in SUMMIT study materials may contact the Study PI.

## Abbreviations

BA: Behavioral activation
DSMB: Data and Safety Monitoring Board
EPDS: Edinburgh Postnatal Depression Scale
GAD-7: Generalized Anxiety Disorder-7 Scale
HAP: Health Activity Program
IRB: Institutional Review Board (US)
NSP: Non-specialist provider
RA: Research assistant
SP: Specialist provider
SH: Sinai Health
SMH: St. Michael’s Hospital
SPIRIT: Standard Protocol Items: Recommendations for Interventional Trials
TMC: Trial Management Committee
UNC: University of North Carolina
WCH: Women’s College Hospital
