# Supplementary material for: Scaling Up Maternal Mental healthcare by Increasing access to Treatment (SUMMIT) through non-specialist providers and telemedicine: a study protocol for a non-inferiority randomized controlled trial
Source: Trials. 2021 Mar 5;22:186. doi: 10.1186/s13063-021-05075-1 (PMC7933917; doi:10.1186/s13063-021-05075-1)
Supplement: Supplementary file 1 — Additional file 1: Figure S1. The Spirit flow diagram: the schedule of enrollment, intervention and assessments. [file 13063_2021_5075_MOESM1_ESM.docx]

Figure S1. The Spirit flow diagram: the schedule of enrollment, intervention and assessments.

|  | **STUDY PERIOD** | | | | | | |
| --- | --- | --- | --- | --- | --- | --- | --- |
|  | **Enrolment** | **Allocation** | **Intervention** | **Post-allocation** | | | **Post child birth** |
| **TIMEPOINT**** | **Pre-allocation** | ***Randomization*** | ***Treatment*** | ***t_1_ (3-month)*** | ***t_2_ (6-month)*** | ***t_3_ (12-month)*** | ***9-15 month post child birth*** |
| **ENROLMENT:** | | | | | | | |
| **Eligibility screen** | X |  |  |  |  |  |  |
| **Informed consent** | X |  |  |  |  |  |  |
| **Baseline assessment** | X |  |  |  |  |  |  |
| **Allocation** |  | X |  |  |  |  |  |
| **INTERVENTION:** | | | | | | | |
| ***Behavioral Activation*** |  |  | X |  |  |  |  |
| **Assessments**: | | | | | | | |
| **Primary outcome** |  |  |  |  |  |  |  |
| ***Depressive symptoms*** | X |  |  | X |  |  |  |
| **Secondary and Other outcomes** |  |  |  |  |  |  |  |
| ***Maternal*** |  |  |  |  |  |  |  |
| ***Demographics (i.e. ethnicity, education, marital status, income)*** | X |  |  |  |  |  |  |
| ***Treatment preference*** | X |  |  |  |  |  |  |
| ***COVID-19 exposure*** | X |  | X | X | X | X |  |
| ***Anxiety symptoms*** | X |  |  | X | X | X |  |
| ***Perceived support*** | X |  |  | X | X | X |  |
| ***Patient-reported activation*** | X |  |  | X | X | X |  |
| ***Disability assessment*** | X |  |  | X | X | X |  |
| ***Quality of life assessment*** | X |  |  | X | X | X |  |
| ***Therapeutic alliance*** |  |  |  | X |  |  |  |
| ***Health service utilization*** | X |  | X | X | X | X |  |
| ***Clinical severity*** | X |  |  | X | X | X |  |
| ***Treatment*** |  |  |  |  |  |  |  |
| ***Therapy quality*** |  |  | X |  |  |  |  |
| ***Session-wise depression/anxiety*** |  |  | X |  |  |  |  |
| ***Patient satisfaction*** |  |  |  | X |  |  |  |
| ***Homework adherence*** |  |  | X |  |  |  |  |
| ***List of medications*** |  |  | X |  |  |  |  |
| ***Child-related*** |  |  |  |  |  |  |  |
| ***Birth weight & length*** |  |  |  |  |  |  | X |
| ***Breastfeeding*** |  |  |  |  |  |  | X |
| ***Psychosocial stimulation*** |  |  |  |  |  |  | X |
| ***Child mental development*** |  |  |  |  |  |  | X |
